# Delaying sexual onset: outcome of a comprehensive sexuality education initiative for adolescents in public schools

**DOI:** 10.1186/s12889-021-11388-2

**Published:** 2021-07-21

**Authors:** Dolores Ramírez-Villalobos, Eric Alejandro Monterubio-Flores, Tonatiuh Tomás Gonzalez-Vazquez, Juan Francisco Molina-Rodríguez, Ma. Guadalupe Ruelas-González, Jacqueline Elizabeth Alcalde-Rabanal

**Affiliations:** 1 Center for Health Systems Research, National Institute of Public Health, Avenida Universidad 655, Colonia Santa María de Ahuacatitlán, 62100 Cuernavaca, Morelos Mexico; 2 Center for Health and Nutrition Research, National Institute of Public Health, Avenida Universidad 655, Colonia Santa María de Ahuacatitlán, 62100 Cuernavaca, Morelos Mexico; 3 Center for Evaluation and Survey Research, National Institute of Public Health, Av. Universidad 655, Colonia Santa María, 62100 Cuernavaca, Morelos México

**Keywords:** Adolescent, Sex education, Sexual debut, School teacher, Sexual behavior, Mexico

## Abstract

**Background:**

A common risk behavior in adolescence is the early initiation of unprotected sex that exposes adolescents to an unplanned pregnancy or sexually transmitted infections. Schools are an ideal place to strengthen adolescents’ sexual knowledge and modify their behavior, guiding them to exercise responsible sexuality. The purpose of this article was to evaluate the knowledge of public secondary school teachers who received training in comprehensive education in sexuality (CES) and estimate the counseling’s effect on students’ sexual behavior.

**Methods:**

Seventy-five public school teachers were trained in participatory and innovative techniques for CES. The change in teacher knowledge (*n* = 75) was assessed before and after the training using t-tests, Wilcoxon ranks tests and a Generalized Estimate Equation model. The students’ sexual and reproductive behavior was evaluated in intervention (*n* = 650) and comparison schools (*n* = 555). We fit a logistic regression model using the students’ sexual debut as a dependent variable.

**Results:**

Teachers increased their knowledge of sexuality after training from 5.3 to 6.1 (*p* < 0.01). 83.3% of students in the intervention school reported using a contraceptive method in their last sexual relation, while 58.3% did so in the comparison schools. The students in comparison schools were 4.7 (*p* < 0.01) times more likely to start sexual initiation than students in the intervention schools.

**Conclusion:**

Training in CES improved teachers’ knowledge about sexual and reproductive health. Students who received counseling from teachers who were trained in participatory and innovative techniques for CES used more contraceptive protection and delayed sexual debut.

## Background

Adolescence is the stage in which reproductive capacity is developed, identity is affirmed, independence is built, and self-assertion is strengthened [1]. During adolescence, life plans are established, but behavioral patterns may represent health risks. One of the patterns is the early debut of unprotected sex that exposes the adolescent to an unplanned pregnancy or sexually transmitted infection (STI) [2]. According to the World Health Organization (WHO), teenage pregnancy is a public health problem, which has negative effects such as: 1) school dropout, 2) abuse of children raised by adolescents, and 3) limited academic and/or job growth; these factors often serve to perpetuate the cycle of poverty [3–6].

The WHO reported in 2012 that around sixteen million teenagers worldwide between the ages of 15 and 19 give birth each year. The children of teenage mothers represent 11% of all births, of which 95% occur in low and middle-income countries [7]. In 2016, Mexico ranked first in adolescent pregnancies (ages 15–19) among members of the Organization for Economic Cooperation and Development (OECD). Mexico’s birth rate of 64.2 per thousand adolescents is much higher than the rest of the member countries [5, 8]. Consequently, the sexual and reproductive health of the adolescent population is a national priority. To address this problem, the Mexican government launched the National Strategy for the Prevention of Adolescent Pregnancy (ENAPEA) in 2015 [9]. ENAPEA aims to reduce births in girls aged 10–14 to zero and to decrease the fertility rate of adolescents aged 15–19 by 50% by 2030. The national average for teenage pregnancy in 2016 was 35 per 1000, with high variability between states. Morelos is a state located in the center of the country near Mexico City and it has one of the highest teen pregnancy rates (36.2 per 1000 adolescents) [10].

Previous research shows that high school students have little knowledge and low perception of the risks and consequences of unprotected sexual practices. Early sexual debut (SD) is a risk factor for adolescent pregnancy and sexually transmitted diseases [11]. International recommendations support the need for comprehensive sexuality education (CSE) programs for adolescents. These programs aim to strengthen knowledge, attitudes and skills in seven areas: gender, sexual and reproductive health, sexual citizenship, pleasure, violence, diversity and interpersonal relationships. Their implementation has been associated with improved knowledge in sexual and reproductive health and fewer risky practices that result in pregnancy and sexually transmitted infections [12, 13]. On the other hand, proper sexual education has been shown to delay sexual initiation, reduce the risk of teenage pregnancies, the frequency of sexual intercourse, the number of sexual partners, and increase the use of condoms and other contraceptive methods [14–16].

Schools are an ideal place to strengthen adolescents’ knowledge and modify their behavior, guiding them to exercise responsible sexuality [17]. It has been documented that teachers who are trained in sex education can act as agents of change and provide students with good quality information, which in turn helps prevent reproductive risk behaviors [18–21]. Research shows encouraging results of sex education interventions that have a multidisciplinary perspective, focus on sexual and reproductive rights, and involve teachers, adolescents, and parents [13–15]. In Mexico, as in other parts of the world, sexual education initiatives for adolescents have been developed in schools but face challenges, such as: teachers’ inadequate knowledge of sexuality issues and limited skills for addressing these topics; occasional educational content that does not match students’ concerns and needs; as well as resistance from parents and educational authorities [22]. Given these problems, it is important to support initiatives for sexual education among adolescents and measure their results. This article aims to assess the knowledge of public secondary school teachers in Morelos, Mexico who received sexual education training and estimate the effect of counseling on students’ sexual behavior.

## Materials and methods

### Description of the intervention

The training model is based on best practices for a Comprehensive Sexuality Education. CSE is built on a framework of rights; it aims to provide adolescents with knowledge, skills, attitudes and values that allow them to enjoy their physical and emotional sexuality on an individual level and in their relationships. CSE views sexuality in a holistic manner, as an integral part of adolescents’ emotional and social development. It recognizes that information alone is not enough; sexual education should provide the opportunity to acquire essential life skills and develop positive attitudes and values towards sex [23]. CSE was implemented in Mexican public schools in two stages. The first focused on teachers and the second on students.

The first stage consisted of two phases. In the first phase, we defined objectives, designed the content and prepared evaluation instruments. In the second phase, teachers were invited to participate in training through the Institute of Basic Education of the State of Morelos (IEBEM). The training workshop was held to improve teachers’ knowledge and skills in CSE for adolescents. The workshop lasted 3 days and focused on four theoretical-methodological axes, which are defined by the following concepts and content: 1) Gender perspective, which distinguishes the differential characteristics, attitudes and behaviors that society attributes to men and women that must be recognized in order to achieve equity [24] (Gender and its expressions in the community, expectations and life-plans, gender inequalities, empowerment, assertive communication); 2) Adolescence and sexuality, which refers to the period of life between 10 and 19 years when sexuality is explored [25] (sexual debut, mythos in sexuality, sexually transmitted infections, Internet and appropriate information sources); 3) Teenage pregnancy and responsible sexuality, which refers to pregnancies during ages 10 to 19 and the responsibility that adolescents must assume when exercising their sexuality [26] (anatomy of pregnancy, implications of teenage pregnancy, sexual self-care); and 4) Teenage contraceptive methods, which focuses on adolescents’ right to know about contraceptive methods and how to use them [12] (contraceptive methods, advantages and disadvantages). The workshop was developed using participatory and innovative methodology with a Gestalt philosophy that included reflection and discussion of each topic [25]. On the basis of the teachers’ tacit knowledge (knowledge embedded in the human mind through experience and jobs) [26] in each theme, a reflective process was carried out and misconceptions and myths were identified. A technique was developed to facilitate teacher-student communication, so that the teacher could learn how to use it and replicate it in class. The workshop facilitators were expert researchers in the subject, knowledgeable about assertive communication skills, and had work experience with teenagers. At the end of the workshop, each teacher was given a kit of materials (electronic folder with the themes developed in the workshop, a flip chart, a poster and leaflets).

The second stage also had two phases. In the first phase, the trained teachers selected the order in which the themes they learned in the workshop (from all four theoretical-methodological axes) would be taught in the classroom (35–40 adolescents from second and third secondary grade). All the topics were addressed in 24 sessions. The methodology employed in each session was diverse, using questions that adolescents proposed and cases that described their sexuality problems, as well as theatrical performances or fairs. Regardless of the technique used, each topic began with a reflection process to recognize positive and negative aspects. Each discussion developed according to the adolescents’ knowledge, while teachers clarified erroneous ideas and myths. To close, teachers and students identified healthy behaviors they should adopt. The teachers covered the themes in the classroom for an average of 8 months, in weekly sessions of 1 h (a total of 24 sessions). In the second phase, the evaluation was performed. At the end of the school year, students who received CES in intervention schools and students from comparison schools were selected to answer a questionnaire. The comparison schools used traditional public-school sex education (TSE) [27], which is requiered for all students in all schools in Mexico. Exceptions are only made for students whose parents have requested exemption due to cultural or religious reasons. The themes in the school curriculum are adjusted according to grade level, although the topics are discussed at the teacher’s discretion. Classes are usually given 1 h a week for an average of 8 months. The themes are oriented towards the anatomy of sexual organs and the use of contraceptives.

### Population and sample

The intervention was designed for teachers and students in second and third grade in public secondary schools in Morelos, Mexico. It was carried out during October 2015–June 2016. For the intervention, 45 schools were randomly selected and 45 for comparison schools. Technical secondary schools are similar to general secondary schools; however, technical secondary emphasizes technological education, according to the economic activity of each region (agriculture, fishing, forestry or services), both in rural and urban communities. Tele secondary is an educational option for communities of less than 2500 inhabitants.

**Fig. 1:**
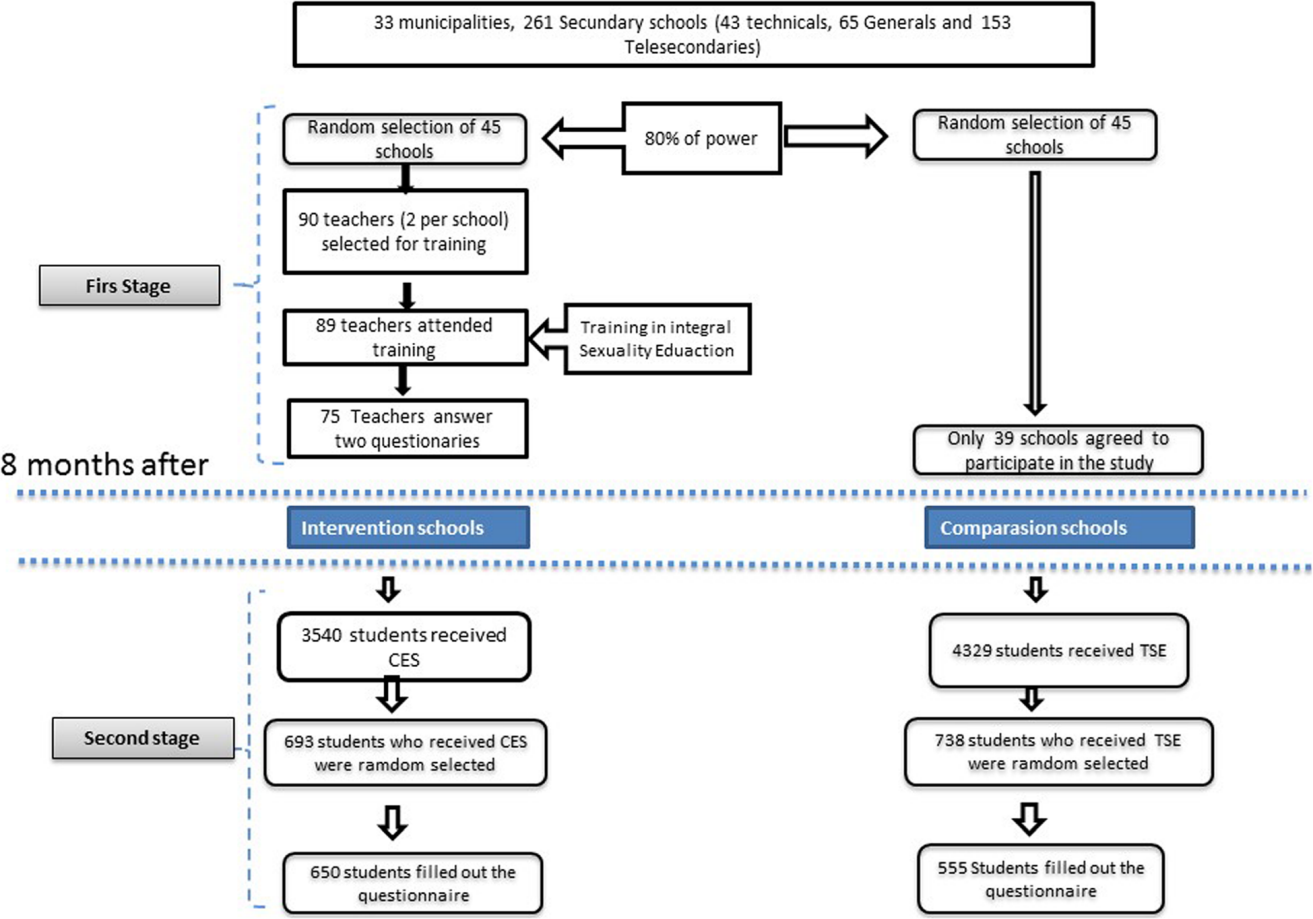
Selection of the study population

To participate in training of CSE, two teachers who taught sex education were randomly selected from each intervention school. The sample of students who received training in CSE was estimated at 693 (from 3540 students in intervention schools) and for students who received TSE, 738 (4329 students from comparison schools). The questionnaires were answered by randomly selected students in both intervention and comparison schools (Fig. 1).

### Outcomes

For teachers, the outcome was knowledge of comprehensive sexuality education, which includes knowledge of gender, adolescence, pregnancy prevention, contraceptive use and sexually transmitted diseases. For adolescents, the outcome was sexual debut, which was measured by self-report of their first sexual intercourse.

### Evaluation design

The change in the knowledge of the trained teachers was evaluated before and after the workshop. We used the questionnaire by the Mexican Foundation for Family Planning, made up of 22 questions [28]. It explored the perspective of gender equality, adolescence and sexuality, teenage pregnancy, responsible sexuality and contraceptive methods. Additionally, it included sociodemographic information like age, sex, the teacher’s main duty (teaching, principal or assistant principal), and type of school (general, technical or tele secondary). The answers to the questions were multiple choice and only one answer was correct; where 0 = incorrect and 1 = correct. The score obtained by each teacher was transformed into a 10-point scale; the score for each methodological axis was multiplied by 10 and divided by the maximum possible score of each methodological axis. To estimate the global score, we added up the scores obtained in all methodological axes, multiplied by 10 and divided by the maximum possible score (twenty two). We classified the score between 0 and 5 as: inacceptable, 5.1–6: regular, 6.1–7: acceptable, 7.1–8: very acceptable, and 8 or more: excellent.

For students, to estimate the effect of CSE, we measured sexual debut as 0 = if the first sexual intercourse occurred more than 6 months prior to the time of answering the questionnaire and 1 = if the first sexual intercourse occurred less than 6 months prior. We applied a questionnaire with 20 items that included sociodemographic variables and explored their reproductive knowledge (gender differences, ITS, Knowledge of contraceptive methods, social effect of pregnancy) and sexual behavior (sexual debut, use of contraceptive in the first and last sexual interaction). The questions to explore reproductive knowledge were multiple choice, e.g. what is the recommended method that provides double protection against pregnancy and sexually transmitted infections? 1 = Abstinence, 2 = Intrauterine device, 3 = Condom, 4 = Hormonal method 5 = I don’t know. The questions to explore sexual behavior were dichotomous, e.g. did you use contraceptive methods during your sexual interaction? 1 = yes 2 = not. The instrument was applied at the end of the school year to both intervention and comparison schools after they had received orientation and counseling in sexual education.

### Data collection

Teachers answered the self-administered questionnaire electronically on a computer provided by the research team before and after the workshop. At the end of the school year, the students received the questionnaire in their e-mails. After answering it, their responses were linked to the *google docs* platform. The questionnaires from teachers and students were answered anonymously.

### Analysis of information

Teachers’ overall knowledge was estimated with the sum of correct answers. The average level of knowledge about the four theoretical-methodological axes was also estimated. Descriptive statistics were estimated for all study variables (percentages, means, medians and confidence intervals). To analyze differences by sex, the Cohen Chi^2^ test was used. To estimate the change in teacher knowledge, the paired Student t-test was used when the scores presented a normal distribution. The Wilcoxon rank sum test for paired data was used when the distributions did not have a normal distribution. We fit a Generalized Estimation Equations model with mixed effects to analyze the characteristics associated with the change in the overall rating. The model was adjusted considering the effect of conglomerates at the school level.

Sociodemographic information, knowledge and reproductive behavior was reported for students. To compare the percentages between intervention and non-intervention schools, the Cohen Chi^2^ statistic was used. We fit a logistic regression model using sexual debut as the dependent variable and used age, sex, school grade and type of school as covariates. Robust variance estimators were calculated by adjusting for the cluster effect at the school level.

### Ethical considerations

The Ethics and Research Committee of the National Institute of Public Health of Mexico (record number 767) approved this project and authorized verbal informed consent for all informants. Therefore, we requested verbal consent from teachers, parents of minors (under the age of 18), and adolescents. Only those informants who freely agreed to participate were included in the study.

## Results

### Effect of the intervention on teachers

Of the 89 teachers who attended the CES training workshop, 84% (75) participated in both measurements (before and after). The teachers came from 26 municipalities in the state. 36% (27) were women, the mean age was 49 ± 9.9 years and 63% (46) were between 40 and 59 years old. 66% of the teachers worked in general secondary schools and 62.7% (47) were Directors or Deputy Directors who also worked as counselors in sex education in the schools (Table 1).

**Table 1:** Characteristics of the teachers involved in training

	N (75)	%	(95%CI)
**Sex**			
Male	48	64.0	(52.9, 75.1)
Female	27	36.0	(24.9, 47.1)
**Age groups**			
19 to 29	1	1.4	(0.0, 4.1)
30 to 39	13	17.8	(8.8, 26.8)
40 to 49	20	27.4	(16.9, 37.9)
50 to 59	26	35.6	(24.4, 46.9)
60 or above	13	17.8	(8.8, 26.8)
**Type of school**			
General secondary	46	64.8	(64.8, 76.2)
Technical secondary	19	26.7	(16.2, 37.3)
Telesecondary schools	6	8.5	(1.8, 15.1)
**Teachers’ duties**			
Directors or Subdirectors	47	62.7	(51.5, 73.9)
Teaching	28	37.3	(26.1, 48.5)

Table 2 shows the scores that the teachers obtained before and after the workshop. Overall, their score before the workshop was 5.1 and afterwards, it was 6.1 out of a total of 10 points. An increase of 0.8 points (*p* < 0.001) was observed in the unadjusted model and 0.9 when the model was adjusted by age, sex, type of school and teacher duties. In general, teachers’ knowledge of adolescence and sexuality, adolescent pregnancy and responsible sexuality and contraceptive methods improved after their participation in the workshop, both in the unadjusted and in the adjusted analysis. (*p* < 0.007).

**Table 2:** Mean scores and differences in adolescents’ sexuality knowledge before and after teacher training

	Mean before	Mean after	Unadjusted difference	Adjusted difference
	(95% CI5%)	(IC95%)	(IC95%)	(IC95%)
Global	5.3 (4.9, 5.6)	6.1 (5.7, 6.4)	0.8*(0.4, 1.2)	0.9* (0.4, 1.3)
Gender perspective	5.2 (4.7, 5.6)	5.7 (5.0, 6.2)	0.5 (−0.2,1.1)	0.7 (0.03, 1.4)
Adolescence and sexuality	5.0 (4.5, 5.5)	5.8 (5.4, 6.2)	0.8* (0.3, 1.4)	0.8*(0.3, 1.4)
Teenage pregnancy and responsible sexuality	4.7 (4.2, 5.2)	5.7 (5.1, 6.3)	1.0* (0.4, 1.6)	1.0*(0.3, 1.7)
Teenage contraceptive methods	6.4 (5.9, 6.8)	7.2 (6.7, 7.8)	0.9* (0.2, 6.5)	0.9 (0.1, 1.6)

Adjusted by: age, sex, type of school and teachers duties

*p* values were adjusted post-hoc using Bonferroni correction *:*p* < 0.035

### Effect of the intervention on students

A total of 1205 students (650 in intervention group and 555 in comparison group) were included to assess the effect of the CSE intervention. The median of age of adolescents in the intervention group was 13.4 and for adolescents in the comparison group, it was 13.8. However, a greater percentage of younger adolescents was observed in the intervention group. Regarding the school grade in the intervention group, there was a higher percentage of students in the second grade, while in the comparison group there was a higher percentage of students in the third grade. Finally, in the intervention group, the majority of the participants were in general secondary schools and in the comparison group, in technical secondary schools (Table 3). 89.4% of students in the intervention group vs. 81.1% in the comparison group responded that they received pregnancy prevention advice. Regarding the effects of pregnancy on adolescents, 84.5% of participants in the intervention group reported they would consider dropping out of school in case of pregnancy and in the comparison group, 79.1%. About 2% of participants in the intervention group reported their sexual debut was (on average) at 14.1 ± (1.5) years, while in the comparison group 5.4% started their sexual debut at 13.1 ± (0.7) years; these differences were statistically significant (*p* < 0.01) (Table 3).

**Table 3:** Characteristics of students in intervention and comparison schools. Morelos, 2016

	INTERVENTION GROUP		COMPARISON GROUP		
	*n* = 650		*n* = 555		
a) **Main social demographic characteristics**					
**Sex**	%	(IC95%)	%	(IC95%)	*p* value^b^
Male	50.5	(46.6 54.3)	46.8	(42.6, 51.0)	
Female	49.5	(45.6, 53.3)	53.2	(48.9, 57.3)	0.211
**Age**					
12 to 13	52.6	(48.7, 56.4)	33.3	(29.3, 37.2)	
14 to 15	46.3	(42.4, 50.1)	66.5	(62.5, 70.4)	
16 to 17	1.1	(0.2, 1.8)	0.2	–	< 0.001
**Grade level**					
Second	54.5	(50.6, 58.3)	42.9	(38.7, 47.0)	
Third	45.5	(41.6, 49.3)	57.1	(52.9, 61.2)	< 0.001
**Type of school**					
General secondary	93.2	(91.2, 95.1)	35.9	(31.8, 39.8)	
Technical secondary	6.8	(4.8, 8.7)	38.9	(34.8, 42.9)	
Telesecondary	0	–	25.2	(21.6, 28.8)	–
**b) Reproductive behavior**					
**Sexual debut**					
Yes	2.0	(0.9, 3)	5.4	(3.5, 7.2)	
No	98.0	(96.9, 99)	94.6	(92.7, 96.4)	^c^ < 0.001
**Pregnancy prevention advice**^a,b^					
Yes	89.4	(87, 91.7)	81.1	(77.8, 84.3)	
No	10.6	(8.2, 12.9)	18.9	(15.6, 22.1)	< 0.001
**Pregnant women drop out of school**					
Yes	84.5	(81.6, 87.2)	79.1	(75.7, 82.4)	
No	15.5	(12.7, 18.3)	20.9	(17.5, 24.2)	< 0.001
**Use of contraceptive in first sexual relation**					
Yes	63.6	(29.7, 97.5)	66.7	(48.7, 84.5)	
No	36.4	(2.4, 70.2)	33.3	(15.4, 51.2)	0.99
s/d	4.0				
**Use of contraceptive in last sexual relation**					
Yes	83.3	(69.1, 97.4)	53.8	(22.4, 85.2)	0.061
No	16.7	(2.5, 30.8)	46.2	(14.7, 77.5)	

^a^ Pregnancy prevention advice includes: pregnancy prevention, use of contraceptive methods and life project

post-hoc unadjusted *p* values; ^b^ Cohen ji^2^; ^c^ Fisher exact test

With respect to the place where they got a contraceptive method, 38.4% (462) of the adolescents reported that they could only acquire them in health centers, 24.8% (299) in pharmacies, 32.4% (390) in health centers and pharmacies, and the remaining (4.4%) obtained contraceptive methods at school, with their parents, with their partner, or they did not specify. There were no statistically significant differences between the comparison and intervention group. 83.3% of participants used a contraceptive method in their last sexual relation in the intervention group and in the comparison group, it was 58.3%.

The ratio of data of the SD as an indicator of reproductive risk was estimated (Table 4). It was found that students in the comparison group had a higher risk of starting sex life earlier compared to the intervention group (OR = 4.7).

**Table 4:** Sexual debut and associated factors in intervention and comparison schools. Morelos, México 2016

	OR	(95%CI)
**Sexual debut**		
Intervention schools	1	
Comparison schools	4.77**	(2.41, 9.43)
**Type of school**		
General secondary	1	
Technical secondary	0.48*	(0.25, 0.94)
Others	0.4	(0.1, 1.56)
**Sex**		
Female	1	
Male	0.5	(0.21, 1.19)
**Age**		
12 to 13	1	
14 to 15	0.77	(0.35, 1.68)
16 to 17	11.29*	(1.54, 82.55)
**Grade level**		
Second	1	
Third	2.31*	(1.02, 5.19)

*p* values were adjusted post-hoc using Bonferroni correction *:*p* < 0.01

Adjusted model by all variables presented in the table

## Discussion

Results from the evaluation of the CSE training model demonstrated that teachers who participated in the workshop increased their knowledge of sexual education. Among the students, there was a significant reduction in SD among those who received sex education from the teachers in the intervention schools vs. the students from the schools in the comparison group.

To strengthen sex education in schools, teachers should be trained in CES to promote adequate knowledge of adolescent sexual health and facilitate teacher-student interactions [29]. It has been documented that sex education in schools in Mexico focuses on a biological approach and that CSE is not sufficiently and adequately addressed in the curricula, plus a lack of teacher training [27]. The Kirby study showed that many issues related to SD in adolescents are not covered by the teacher in the classroom, which is why training is needed to prepare teachers as facilitators in sex education [17, 30]. Currently, traditional and conservative norms and pedagogical practices are imposed in school sex education programs [17]. Implementing sexuality-related educational strategies with adolescents through teachers is a challenge [31].

Several studies have shown that school training interventions that improve teachers’ skills in sexual health maximize the effectiveness of interactions with their students. These interventions have shown results in reducing risky sexual behaviors and preventing teenage pregnancy [32]. Furthermore, CSE is effective in influencing adolescents’ decisions, such as delaying sexual debut [23]. Therefore, training teachers in sex education is a strategy that is recommended worldwide, but its development and implementation is still limited [33]. It is interesting to note that the training offered to teachers in this intervention included topics related to STIs and showed positive results in their knowledge improvement, despite the short period of training. These results could be attributed to the use of a reflective methodology and the teacher’s recovery of tacit knowledge, which they could have applied to the subject [34]. It is also important to highlight that young people identify different actors to meet their reproductive health needs; from parents as confidants in courtship issues, to doctors for sexuality problems (sexual impotence and pregnancy), and to teachers as counselors in sexuality issues [35].

Likewise, the evidence shows that STIs occur at an earlier age and that the risk perception is non-existent for adolescents [36]. Therefore, it exposes adolescents to having a greater number of sexual partners which is associated with unsafe sexual practices and carries greater risks of contracting STIs [37, 38]. It also exposes them to an unplanned pregnancy that forces them to take responsibility for the care of a child and alters their personal development plans [34]. The results of this study show that adolescents who receive adequate counseling on sexuality will delay SD. Similar studies show that for the programs to be effective and achieve the expected result in sexual behavior, they must address issues related to pregnancy prevention, STIs, HIV / AIDS, encourage contraceptive use and provide tools to cope with peer pressure [39]. These topics were extensively developed in the training model with the teachers of the intervention schools.

The main limitation of this study lies in the design of the evaluation. The ideal effect evaluation design should include before and after measurements of teachers and students in both the intervention and comparison groups. For budgetary reasons, it was not possible to fully implement this design, so the evaluation in teachers was limited to before-after measurements only performed in the group of teachers who received the training. The other limitation is that we do not evaluate teachers’ knowledge and skills in CSE at the end of the school year. It is likely that these skills improved, since they had to review the information in order to teach the themes to their students. In the students, a cross-sectional measurement was conducted after the CSE implementation in the intervention and comparison schools. Although schools were randomly selected for both the intervention and comparison groups, there were differences in the types of schools included in each group. Additionally, we cannot rule out that other events outside the intervention (social networks and internet use that were not measured in the study) may have influenced the increase in knowledge. It could also be argued that the differences between intervention and comparison groups (in the case of students) are due to differences in their characteristics. In the case of the students, the analysis was adjusted by characteristics (age, sex and schooling and type of school) to control the effect that the differences between the groups could have. It is possible that students in secondary schools have a greater interest in continuing their studies than those in technical secondary schools and tele schools. They may place more importance on staying in school because it is an important part of their future life plans [27, 40, 41]. Finally, we do not know if teachers and students from the schools that participated in the intervention shared materials with teachers and students from the comparison schools.

## Conclusions and recommendations

Training teachers in issues related to comprehensive sexuality through participatory and reflexive methodology strengthens their knowledge and skills to transmit information to their students in an appropriate manner. In this study, students who received information from teachers who were trained in CSE used more contraceptive protection and delayed SD [27, 29]. Consequently, in light of the results presented, we recommend that schools develop innovative and attractive sex education programs for adolescents as they are ideal settings to implement responsible sexuality programs for this population. Therefore, teachers must be continuously trained in innovative methodology to become sexual education counselors and help students reduce their sexual risk behaviors [28, 32].

## Data Availability

The datasets generated and/or analyzed during the current study are not publicly available since we made an agreement with the Institute of Basic Education of the State of Morelos not to publish the database for free access. It will be used only for academic purposes. For this reason, the data are available from the corresponding author on reasonable request**.**

## Abbreviations

AIDS: Acquired Immune Deficiency Syndrome
CSE: Comprehensive sexuality education
ENAPEA: National Strategy for the Prevention of Adolescent Pregnancy
HIV: Human Immunodeficiency Virus
IEBEM: Institute of Basic Education of the State of Morelos
OECD: Organization for Cooperation and Development Economic
SD: Sexual debut
STI: Sexually transmitted infection
TSE: Traditional sex education
WHO: World Health Organization

## References

[1] Greydanus DE, Bashe P, American Academy of Pediatrics (2003). Caring for your teenager: the complete and authoritative guide.

[2] Guzmán J, Hakkert R, Contreras J, Falconier de Moyano M (2001). Diagnóstico sobre Salud Sexual y Reproductiva de Adolescentes en América Latina y el Caribe.

[3] Jiménez-González A, Granados-Cosme JA, Rosales-Flores RA (2017). Embarazo en adolescentes de una comunidad rural de alta marginalidad. Un estudio mixto de caso. Salud Publica de Mexico.

[4] Organización Panamericana de la salud (OPS) (2018). Aceleración mundial de las medidas para promover la salud de los adolescentes: Orientación para la aplicación en los países.

[5] Quiroz J, Atienzo EE, Campero L, Suárez-López L (2014). Entre contradicciones y riesgos: Opiniones de varones adolescentes Mexicanos sobre el embarazo temprano y su asociación con el comportamiento sexual. Salud Publica de Mexico.

[6] Stern, C. El embarazo en la adolescencia como problema público: Una visión crítica. Salud Publica de Mexico. 1997;56(2):180–8. Salud Publica de Mexico. 39(2):137–43. 10.1590/s0036-36341997000200008. 9254438

[7] Organización Mundial de la Salud. Prevenir el embarazo precoz y los resultados reproductivos adversos en adolescentes en los países en desarrollo: las evidencias. (2012) http://apps.who.int/iris/bitstream/10665/78253/1/WHO_FWC_MCA_12_02_spa.pdf.

[8] Fondo de Población de las Naciones Unidas (UNFPA) (2014). Directrices operacionales del UNFPA para la educación integral de la sexualidad.

[9] Gobienro de la Republica de México. Estrategia Nacional para la Prevención del Embarazo en Adolescentes. 2015. https://www.gob.mx/cms/uploads/attachment/file/232826/ENAPEA_0215.pdf

[10] Morelos S, Salud SS (2018). Análisis de la Situación de Salud y de la Respuesta Social Organizada de la Población sin Derechohabiencia del Estado de Morelos.

[11] Odeigah L, Rasaki SO, Ajibola AF, Hafsat AA, Sule AG, Musah Y (2019). High risk sexual behavior among adolescent senior secondary school students in Nigeria. Afr Health Sci.

[12] IPPF (2010). IPPF framework for comprehensive sexuality education.

[13] Kirby D (1993). Sexuality education: it can reduce unprotected intercourse. SIECUS Rep.

[14] Haignere CS, Culhane JF, Balsley CM, Legos P (1996). Teachers’ receptiveness and comfort teaching sexuality education and using non-traditional teaching strategies. J School Health.

[15] Gayet C, Rosas CA, Magis C, Uribe P (2002). Con quién hablan los adolescentes mexicanos sobre el SIDA Whom do Mexican adolescents talk to about AIDS?. Salud publica de Mexico.

[16] UNESCO (2015). Comprehensive sexuality education in teacher training in Eastern and Southern Africa.

[17] Kirby D (2002). The impact of schools and school programs upon adolescent sexual behavior. J Sex Res.

[18] Clara M, Restrepo A, Ángel J, Roberto J, Manzanero L, Sabina E, Llanio LQ (2010). La Formación Docente en Educación de la Sexualidad en América Latina y el Caribe.

[19] Juárez F, Gayet C (2005). Salud sexual y reproductiva de los adolescentes en México: un nuevo marco de análisis para la evaluación y diseño de políticas. Papeles de población.

[20] Lindau ST, Tetteh AS, Kasza K, Gilliam M (2008). What schools teach our patients about sex: content, quality, and influences on sex education. Obstet Gynecol.

[21] Nguyen G, Costenbader E, Plourde KF, Kerner B, Igras S. Scaling-up normative change interventions for adolescent and youth reproductive health: an examination of the evidence. J Adolescent Health. 2019;64(4S):S16–30. 10.1016/j.jadohealth.2019.01.004 21. 10.1016/j.jadohealth.2019.01.004 PMC6426721 30914164

[22] Rojas R, de Castro F, Villalobos A, Allen-Leigh B, Romero M, Braverman-Bronstein A, Uribe P (2017). Comprehensive sexual education in Mexico: an analysis of coverage, comprehensiveness and continuity of contents in Mexican public and private schools. Salud Publica de Mexico.

[23] Organización de las Naciones Unidas para la Educación, la Ciencia y la Cultura (2014). Educación Integral de la Sexualidad: conceptos, enfoques y competencias.

[24] Ravalli MJ, Calisti NL (2017). Perspectiva De Género. ¿De qué hablamos cuando hablamos de perspectiva de género? Fondo de las Naciones Unidas para la Infancia (UNICEF).

[25] López PJ, Martínez Galván A, I. y López Corral J (2013). La sexualidad en las etapas de la vida. Características. Principales conflictos y sus afrontamientos.

[26] Becerril-Montekio V, Alcalde-Rabanal J, Darney BG, Orozco-Nuñez E (2016). Using systematized tacit knowledge to prioritize implementation challenges in existing maternal health programs: implications for the post MDG era. Health Policy Plann..

[27] Fondo de Naciones Unidas. Estado de la formación docente en educación de la sexualidad, salud sexual y reproductiva en América Latina y el Caribe. Diagnóstico de los Estados Unidos Mexicanos. 2005

[28] Rodríguez RG, Aguilar Gil JA (2009). Hablemos de sexualidad con la gente joven : modelo educativo para profesores y profesionales que trabajan con jóvenes.

[29] Goldman JD (2011). An exploration in health education of an integrated theoretical basis for sexuality education pedagogies for young people. Health Educ Res.

[30] Thammaraksa P, Powwattana A, Lagampan S, Thaingtham W (2014). Helping teachers conduct sex education in secondary schools in Thailand: overcoming culturally sensitive barriers to sex education. Asian Nurs Res.

[31] Oliveira-Campos M, Giatti L, Malta D, Barreto SM (2013). Contextual factors associated with sexual behavior among Brazilian adolescents. Ann Epidemiol.

[32] Voisin DR, Salazar LF, Crosby R, Diclemente RJ, Yarber WL, Staples-Horne M (2005). Teacher connectedness and health-related outcomes among detained adolescents. J Adolescent Health.

[33] Demaria LM, Galárraga O, Campero L, Walker DM (2009). Educación sobre sexualidad y prevención del VIH: un diagnóstico para América Latina y el Caribe Sex education and HIV prevention: an evaluation in Latin America and the Caribbean. Rev Panamericana de salud publica.

[34] LaChausse RG, Clark KR, Chapple S (2014). Beyond teacher training: the critical role of professional development in maintaining curriculum fidelity. J Adolescent Health.

[35] Mason-Jones AJ, Sinclair D, Mathews C, Kagee A, Hillman A, Lombard C (2016). School-based interventions for preventing HIV, sexually transmitted infections, and pregnancy in adolescents. Cochrane Database Syst Rev.

[36] Centro Nacional para la prevención y el control del VIH/SIDA & Instituto Nacional de Salud Pública (2014). Análisis sobre educación sexual integral, conocimientos y actitudes en sexualidad en adolescentes escolarizados.

[37] Chacón-Quesada T, Corrales-González D, Garbanzo-Núñez D, Gutiérrez-Yglesias JA, Hernández-Sandí A, Lobo-Araya A, Romero-Solano A, Sánchez-Avilés L, Ventura-Montoya S (2009). ITS Y SIDA en adolescentes: descripción, prevención y marco legal. Medicina Legal de Costa Rica.

[38] Secor-Turner M, Kugler K, Bearinger LH, Sieving R (2009). A global perspective of adolescent sexual and reproductive health: context matters. Adolescent Med.

[39] Fonner VA, Armstrong KS, Kennedy CE, O'Reilly KR, Sweat MD (2014). School based sex education and HIV prevention in low- and middle-income countries: a systematic review and meta-analysis. PLoS One.

[40] Lorenzo O, Zaragoza J. Secondary and Higher Education in Mexico: theoretical analysis of the current situation. Dialnet. 2014;6:59–72. https://dialnet.unirioja.es/servlet/articulo?codigo=4733974.

[41] Secretaría de Educacion Pública. La estructura del Sistema educativo mexicano. 2018. https://siteal.iiep.unesco.org/sites/default/files/sit_accion_files/siteal_mexico_0101.pdf

